# In Reply: Immune checkpoint inhibition in medulloblastoma: moving toward biologically informed strategies

**DOI:** 10.1093/oncolo/oyag387

**Published:** 2026-10-01

**Authors:** Marta Viana-Pereira, Patrícia Fontão, Rui Manuel Reis

**Affiliations:** Life and Health Sciences Research Institute (ICVS), School of Medicine, University of Minho, Campus de Gualtar, Braga, 4710-057, Portugal; Oncology Department, Hospital de Braga, Sete Fontes, Braga, 4710-243, Portugal; Life and Health Sciences Research Institute (ICVS), School of Medicine, University of Minho, Campus de Gualtar, Braga, 4710-057, Portugal; Life and Health Sciences Research Institute (ICVS), School of Medicine, University of Minho, Campus de Gualtar, Braga, 4710-057, Portugal; Molecular Oncology Research Center, Barretos Cancer Hospital, Rua Antenor Duarte Villela, 1331, Barretos, S. Paulo, 14784 400, Brazil

Dear Editor,

We thank Wang et al.^1^ for their thoughtful response to our systematic review and for sharing their experience with immune checkpoint inhibitor (ICI) maintenance following intensive upfront therapy and tandem autologous hematopoietic cell transplantation (HCT) in children with high-risk medulloblastoma. Their observations add an important, clinically distinct perspective to the heterogeneous studies compiled in our review, specifically on using anti-PD-1 therapy as maintenance following intensive multimodal therapy and tandem autologous HCT.

Immunotherapy in the studies included in our review was predominantly investigated in patients with recurrent or refractory setting but was not restricted to patients with measurable disease or exclusively administered as treatment for active relapse.^2^ Nevertheless, the experience reported by Wang et al. is distinct in its use of anti-PD-1 specifically as post-transplant maintenance and provides important additional information in this setting.

The toxicity reported by Wang et al. in these three patients is noteworthy, particularly because two developed grade 3 pneumonitis, without disease benefit attributable to ICI therapy. These observations add valuable information to the literature, given the limited medulloblastoma-specific toxicity data previously reported. Nevertheless, the very small number of patients and the complexity of preceding treatment, including craniospinal irradiation, intensive chemotherapy, and tandem HCT, preclude attributing this toxicity specifically to the post-transplant use of ICI. These findings underscore the importance of prospective, setting-specific safety reporting, particularly when immunotherapy is incorporated after intensive multimodal treatment.

The limited clinical activity of ICI identified in our systematic review, together with the observations reported by Wang et al. raises a broader question: which patients with medulloblastoma may benefit from ICI? Among the currently recognized biological contexts, constitutional mismatch repair deficiency (cMMRD) is arguably one of the most compelling candidates for ICI sensitivity, since mismatch-repair deficiency can generate an ultrahypermutated phenotype and high neoantigen burden.^3^ Nevertheless, the number of medulloblastoma patients treated in this context remains very small, and cMMRD cannot yet be regarded as a clinically validated predictive biomarker of ICI response in medulloblastoma.^3^^,^^4^

Moreover, emerging evidence highlights medulloblastoma’s biological and immunological heterogeneity. Recent transcriptomic analyses suggest that distinct molecular subgroups harbor different patterns of T-cell activity and exclusion, potentially identifying tumors with greater susceptibility to immunotherapeutic approaches.^5^ Complementary single-cell analyses have further demonstrated subtype-specific immune architectures.^6^ These findings remain hypothesis-generating and require prospective clinical validation, but they argue against considering medulloblastoma as an immunologically uniform disease. A recent review provides an updated overview of ongoing clinical trials evaluating immune checkpoint inhibition in medulloblastoma.^7^

Taken together, Wang et al.'s^1^ observations and emerging biological data suggest that future investigation should address not only when checkpoint inhibition is administered, but also which tumors and immune contexts are most likely to benefit from these treatments. Prospective trials incorporating molecular characterization, immune profiling, and setting-specific toxicity assessment will be essential to determine whether ICI has a meaningful role in selected patients with medulloblastoma.
